# Pulmonary Embolism Secondary to Intravenous Immunoglobulin in a Child with Leukemia

**DOI:** 10.4274/tjh.galenos.2021.2021.0400

**Published:** 2021-12-07

**Authors:** Işıl Seren Oğuz, Zühre Kaya, Serap Kirkiz, Ülker Koçak

**Affiliations:** 1 Gazi University Faculty of Medicine, Department of Pediatrics, Ankara, Turkey; 2 Gazi University Faculty of Medicine, Unit of Pediatric Hematology-Oncology, Ankara, Turkey

**Keywords:** Immunoglobulin, Pulmonary embolism, Leukemia, Children

## To the Editor,

Pulmonary embolism (PE) secondary to intravenous immunoglobulin (IVIG) is a rare life-threatening complication that occurs in 1% of patients with hematologic malignancies [1]. This complication has mainly been described in adults with chronic lymphocytic leukemia and multiple myeloma [1]. To our knowledge, there have been no previous reports of PE resulting from IVIG administration in a child with acute lymphoblastic leukemia (ALL).

Our patient was an 11-year-old boy with high-risk ALL who was treated with the ALL-BFM-95 protocol, as described previously [2]. Since the patient had an HLA-matched sibling donor, we planned a third high-dose chemotherapy regimen followed by allogeneic stem cell transplantation (allo-SCT). Hypogammaglobinemia was detected prior to allo-SCT. The IVIG preparation was administered at 400 mg/kg (total dose: 20 g). The product information for this IVIG advises a slow infusion at 0.3 mL/kg/h for the first 30 min, to gradually increase to 4.8 mL/kg/h if no reaction occurs and to be completed within 4 h.

**Figure 1:**
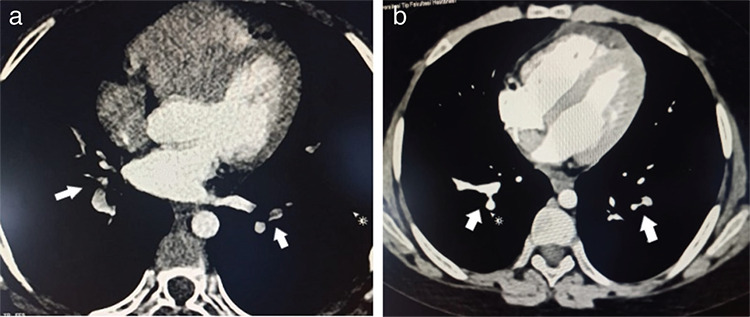
a) Partial filling defects in the segmental and subsegmental branches of the pulmonary artery in the lower lobe of both lungs. b) Resolution of thrombus in the segmental and subsegmental branches of the pulmonary artery in the lower lobe of both lungs.

Approximately 1.5 h after our patient’s infusion ended, he developed shortness of breath and oxygen desaturation (SpO_2_ 92%). He had no fever or hypotension, and chest radiography was normal. Complete blood count results were within normal limits, but the patient’s D-dimer level was slightly elevated at 0.67 mg/L (normal: <0.5 mg/L). Oxygen support was initiated and, although there was no fever, we ordered a COVID-19 PCR test. The PCR test was negative, but when the patient’s blood oxygen level did not improve during follow-up, chest computed tomography angiography was performed. Partial filling defects consistent with thrombus were observed in the segmental and subsegmental branches of the pulmonary artery, in the lower lobes of both lungs (Figure 1a). The patient was diagnosed with PE. Low-molecular-weight heparin was initiated, with 100 IU/kg divided into two doses and given subcutaneously. On the first day of treatment, the dyspnea improved. The patient’s oxygen requirements began to decrease on day 2, and on day 3 his SpO_2_ was 98%.

The PE was suspected to be IVIG-induced because the patient was in complete remission at the time of the event and did not have febrile neutropenia, inciting drug therapy, or a central venous catheter. Risk factors for inherited thrombophilia were excluded. Doppler ultrasound for lower extremity thrombosis, echocardiography findings, and levels of antithrombin 3, antiphospholipid antibodies, C3, and C4 were all normal. The PE gradually resolved with heparin during the second week after diagnosis, similar to a recent report (Figure 1b) [3].

The pathophysiologic mechanisms of PE due to IVIG are poorly understood. Some reports have suggested possible contributors to the development of PE in these patients including increased hyperviscosity (i.e., infusion rate not exceeding 200 mL/h or 0.08 mL/kg/min) secondary to rapid infusion, resulting in a hypercoagulable state, and serum complement or platelet activation due to exogenous immunoglobulin G [4,5,6,7]. A comprehensive review noted that most thromboembolic complications occurred within 24 h of IVIG administration [8]. Our experience suggests that leukemia specialists should be aware of the potential for PE complications after IVIG administration in children with leukemia.
